# Dyslexia with and without developmental language disorder: Profile analysis

**DOI:** 10.1007/s11881-025-00358-7

**Published:** 2026-03-03

**Authors:** Margaret J. Snowling, M. Emma Hayiou-Thomas, Charles Hulme

**Affiliations:** 1 University of Oxford and St. John’s College , Oxford, UK; 2 University of York St. John, York, UK; 3 University of York, York, UK; 4 University of Oxford, Oxford Brookes University, Oxford, UK

**Keywords:** Dyslexia, Developmental Language Disorder (DLD), Language difficulties, Comorbidity, 2D model

## Abstract

The characterization of dyslexia as a language learning impairment can be traced back to Samuel Orton who in 1937 wrote that ‘strephosymbolia’ (his term for the condition) was due to an impairment of the ‘language faculty’. Some 40 years later, Vellutino (Theory and research, 1979) published his classic text, Dyslexia: Theory and Research in which he argued that dyslexia could be characterized as a verbal processing deficit. However, in more recent times, the language difficulties of dyslexia have been underplayed Adlof et al. (Language, speech, and hearing services in schools, 49(4):762-773, 2018). First, the discrepancy definition of dyslexia considers it a specific learning disability ‘diagnosed ’ when reading skills are below expectation given IQ, typically ruling out children with lower verbal abilities (Snowling et al., 2020). Second, the phonological deficit hypothesis states that phonological (speech) processing skills are impaired in dyslexia, regardless of IQ Stanovich et al. (In Perspectives on the classification of specific developmental disorders :105-136, 1998) and hence often despite good oral abilities. Within these views, dyslexia is a disorder of written language distinct from developmental language disorder (DLD) in which spoken language skills are impaired. In this paper we take a close look at the similarities and differences between dyslexia with and without DLD and discuss their shared cognitive risk factors, drawing out implications for assessment and intervention.

**Supplementary Information:**

The online version contains supplementary material available at https://doi.org/10.1007/s11881-025-00358-7.

It has long been established that preschool language difficulties are a risk factor for poor reading. In a population study, Rutter and Yule (1975) showed that children with specific reading difficulties/dyslexia had a history of speech and language difficulties. Examining how preschool oral language difficulties affect reading, Catts et al. (2005) reported data from a representative population sample indicating that between 14 and 19% of children with DLD in kindergarten (referred to as specific language impairment (SLI) at the time) went on to be dyslexic in Grades 2,4 and 8. Snowling et al., (2019), following children in a high-risk sample, reported a higher rate: 40% of children with preschool DLD were identified as dyslexic at age 8; similarly elevated risks have been reported in clinical samples (e.g., Conti-Ramsden et al., 2001; McArthur et al., 2000). In line with these findings, a meta-analysis of family risk studies reported significantly poorer language in preschool children who went on to be classified as dyslexic than in typical controls (d = 0.72 for grammar and 0.83 for vocab; Snowling et al., 2016).

 Together these findings raise the question of the relationship between dyslexia and DLD: is there continuity between the disorders (is DLD the antecedent of dyslexia; is DLD a more severe form of dyslexia; or are the disorders comorbid (do the two disorders co-occur)? However, both reading and language disorders are heterogenous (Joanisse et al., 2000; Ramus et al., 2013) and subject to developmental change (Snowling et al., 2016; Scarborough, 1998). More specifically, Bishop and Adams (1990) proposed the critical age hypothesis - if a child’s language difficulties persist at school entry, then the risk of poor reading is high but if difficulties resolve then the child is likely to have a normal reading outcome. In a follow-up study of children from the Bishop and Adams’ sample, Snowling et al. (2000) went on to show that children with preschool DLD (termed specific language impairment in this study) were likely to develop dyslexia in adolescence only if their language difficulties were not resolved by school entry (age 5;06 in this study). Along similar lines, Snowling et al. (2020) showed that, among children with a preschool history of DLD, reading comprehension impairments at age 9 were more likely if they had persistent language impairments at age 5;5 years but not if these were resolved, Thus, the nature of the relationship between dyslexia and DLD will depend not only upon the population that is studied and the diagnostic criteria used but also the age of the children.

Addressing the shared risks between dyslexia and DLD, Bishop and Snowling (2004) proposed a two-dimensional model (hereafter 2D model) According to this model, at the core of learning to read (and the core impairment in dyslexia) are phonological skills; however broader language skills (described as non-phonological) determine whether or not there are problems of reading comprehension, such as those typically associated with DLD. Testing the validity of this model, Catts et al. (2005) reported the outcomes of children in a population sample studied from kindergarten. Findings revealed three distinct clinical groups in Grade 1: dyslexia, DLD-only and DLD with dyslexia; the groups differed in phonological awareness and in nonword repetition (those with dyslexia and dyslexia + DLD were impaired), and, by definition, both groups with DLD were impaired in language skills. Given there were two subgroups of children with DLD, one with dyslexia, one without, they argued that dyslexia and DLD are distinct conditions that frequently co-occur (comorbidity).

Ramus et al. (2013) also tested the 2D model, in this case in a clinical sample of children with DLD or dyslexia aged between 8 and 12 years. In addition to investigating performance on tests of phonological and non-phonological skills, they also investigated performance on assessments of what they referred to as ‘phonological representations’ - measures, such as nonword discrimination, nonword repetition, and articulation which, they argue, require access to high-quality phonological features of words but not operations on these representations (such as would be required by metacognitive or retrieval processes). Exploratory factor analysis of scores on these measures confirmed the validity of three dimensions accounting for a high proportion of variance in linguistic skills: non-phonological language, phonological skills and phonological representations. Proceeding from this they formed three composite measures of the tasks with high loadings on each factor and compared the performance of dyslexic, DLD and DLD + dyslexia groups on these measures. As predicted, the dyslexic groups showed deficits in phonological but not non-phonological skills compared to controls; they did not show deficits in phonological representations (Mundy & Carroll, 2012 for similar findings). In contrast, a DLD-only group were impaired in non-phonological language tasks, while the comorbid group had deficits in all three domains. Thus, referring to the 2D model, both the dyslexia and the DLD + dyslexia groups had difficulties in phonological skills, while both DLD groups had impairments in language skills (similar to Catts et al., op cit) ; since both DLD-only and DLD + Dyslexia groups also had difficulties on tasks tapping phonological representations, they proposed a third dimension in the model. However, it is noteworthy that they reported high correlations between phonological and non-phonological language skills (r = .74) and between phonological skills and measures tapping phonological representations (r = .66). 

Turning to the findings of a study of children at high risk of dyslexia (either because they were at family risk or because of preschool language difficulties), Snowling et al. (2019) reported two different trajectories among children who in the preschool years were identified as having developmental language disorder. One group went on at age 8 to have comorbid dyslexia (DLD + Dyslexia) while the other comprised children who did not reach criteria for dyslexia (DLD-only). To account for these diverse profiles, they proposed two separable risks for poor reading. The first being poor language, its impact on reading mediated by phoneme awareness (Hulme et al., 2015). The second, a specific phonological deficit that affects performance on tasks such as phoneme awareness, rapid naming and letter learning. According to this model the two risks are separable and therefore also additive in the case of comorbidity (DLD + Dyslexia).

 From the clinical perspective, Adlolf and Hogan (2018) argue that it is not unusual for children with dyslexia to experience sub-clinical language difficulties yet it is common for researchers to ignore this co-occurrence. Along similar lines, Stevens et al. (2022) point out that a large proportion of children with dyslexia also experience language comprehension problems.

 Theoretically, they cite the Reading Systems Framework (Perfetti & Stafura, 2014) which proposes that semantic knowledge (at the core of the non-phonological dimension of the 2D model) influences both word reading and reading comprehension. Similarly, Snowling & Hulme (2025) propose that language is at the heart of reading with a direct impact on reading comprehension and an indirect effect on decoding via phoneme awareness.

 Together, these views highlight the importance of longitudinal studies that examine the co-occurrence of poor reading and poor language, and the interrelationship between these skills as they unfold over time. Here we investigate the similarities and differences between children with dyslexia and children with dyslexia + DLD using cases from a longitudinal study of children at risk of reading difficulties followed from age 3;06 years (Nash et al., 2013). As age 8, these children were classified into four outcome groups: dyslexia, DLD, dyslexia + DLD and typical reader (Snowling et al., 2019). Examination of the profiles of these groups over time suggested that phonological impairments were evident in children who developed dyslexia from the preschool years onwards; these were more severe in the dyslexia + DLD group than in the dyslexia-only group. In contrast, the DLD-only group did poorly on phonological measures in preschool but their difficulties lessened after age 5;06, such that they showed better decoding skills than the DLD + dyslexia group throughout the period in which they were followed (and hence did not reach the criterion for dyslexia of −1SD below average in literacy at age 8).The two groups with a DLD outcome showed weak language skills from preschool onwards while the dyslexia outcome group were free of language difficulties in preschool with some showing increasing problems after school entry. Here we report data from a follow-up study approximately one year after the time at which the children were classified according to outcome focusing on the two groups with dyslexic outcomes. In addition to reading, we also include mathematical attainment given the substantial overlap between dyslexia and arithmetic difficulties (Goebel, 2014). 

The study aimed to address the following questions:

1. Do children classified as reaching criteria for ‘dyslexia’ but not DLD do more poorly on non-phonological language tasks, hereafter Language, than typical readers? We hypothesized they would show sub-clinical language impairments (Adlof & Hogan, 2018).
2. Are there similarities between children with Dyslexia and with DLD + Dyslexia on tests tapping phonological skills? Since poor phonological skills are the proximal risk for poor reading, and both groups experienced phonological difficulties from preschool (Snowling et al., 2019) we predicted this would be these case; further we predicted that these would be more severe in the Dyslexia + DLD group given the impact of language on phonological awareness (Hulme et al., 2015).
3. Are there differences in the attainments of Dyslexia and DLD + Dyslexia groups one year following outcome ‘classification’ ? We investigated both reading and mathematical attainments.

## Method

The present paper reports data from the Wellcome Reading and Language Project (Nash et al., 2013), a longitudinal study of a sample of children at high risk of dyslexia either because of an affected relative (family risk) or because of preschool language difficulties (at the time of the study the latter group was described as specific language impairment (SLI)). Ethical clearance for the study was provided by the University of York, Department of Psychology’s Ethics Committee and the NHS Research Ethics Committee. Parents provided informed consent for their child to be involved. Children were assessed at approximately annual intervals from age 3.5 years by experienced researchers who were trained and observed to ensure fidelity. The current study reports data from the assessment at Time 6, one year after the children were classified as reaching the study criteria for Dyslexia at Time 5 (based on performance 1.5 SD below the mean of the low-risk control group on a composite of reading and spelling; SS < 89) or Dyslexia + DLD (below average (−1SD) on a composite language measure formed by averaging the age-standardized scores from *Expressive Vocabulary* (*CELF–4;* TROG–2 and *Recalling Sentences* (Snowling et al., 2019).

## Participants

At Time 6 of the Wellcome Reading and Language Project, 233/260 of the participants remained in the sample (90% retention rate). Their mean age was 109.72 months (SD = 6.18; range 93–125 months). Of these, 18 children were classified as dyslexic, 28 as dyslexia + DLD. The typical reader group comprised 144 children with neither ‘diagnosis’ (regardless of preschool risk status). A further group of children with DLD but not dyslexia (DLD-only) were not included in the present study.

### Assessment measures

####  Language skills

##### Expressive Vocabulary

The *CELF 4 Expressive Vocabulary* test (Wiig et al., 2006) was used to assess expressive vocabulary. The child is shown a series of pictures and asked what is in the picture. Responses are scored 0,1 2 according to the test manual. To avoid ceiling effects the test was extended by adding 8 items, making a total of 35 items (α = 0.66). Testing was discontinued after 7 consecutive scores of 0.

##### Receptive Vocabulary

The *Receptive One Word Picture Vocabulary Test (ROWPVT;* Brownell, 2000) (α = 0.95) is a word: picture matching task. Testing was discontinued after 6 incorrect responses out of 8 consecutive items.

##### Listening Comprehension

The YARC listening comprehension was given as a test of language comprehension. Children hear a story and are asked questions tapping either facts given in the story or inferencing skills. The children heard 2 stories and answered 21 questions (α = 0.74).

#### Phonological Skills

##### Phoneme deletion

In this test, the child had to delete phonemes from items increasing in difficulty. There were 24 items in 3 blocks in which the sounds to be deleted is (i) at beginning or end of a word without clusters (ii) from a cluster at beginning or end of a word (iii) in the middle of the word within clusters. Items were scored correct or incorrect. The blocks were each discontinued after 5 consecutive errors.

##### Rapid Naming (RAN)

This test required the child to name as quickly as possible a set of digits (1–4) displayed at random in a matrix, 8 items per row, 5 rows in total. The time taken to complete the test was recorded and expressed as items per minute.

##### Nonword repetition

In this test, children are asked to repeat a series of 20 nonwords (*funny made up words)* of 1–5 syllables played through an audio-recorder. Responses are scored correct or incorrect.

#### Attainments

##### Reading

Five measures of word-level reading skills were administered. The three subtests from the (untimed) *Diagnostic Test of Word Reading Processes* (Forum for Research in Language and Literacy, 2012): Regular, Exception and Nonword Reading. Testing discontinued after 5 consecutive errors. The reliabilities are: regular words 0.88; irregular words 0.83; nonwords 0.77 (for the composite score α = 0.97). To assess reading fluency, children were asked to read items on the Test of Word Reading Efficiency (TOWRE; Torgersen, Rashotte & Wagner, 1999) which requires the rapid reading of lists of words and nonwords in 45 s (test-retest reliability = 0.93).

##### Reading Comprehension

The child read passages from the *York Assessment of Reading for Comprehension (YARC Passage Reading*) and accuracy was monitored. The child then answered 8 spoken comprehension questions about each passage. Accuracy and comprehension ability scores are calculated based on the two most difficult passages the child read (α = 0.85). The comprehension scores are reported here.

##### Arithmetic

The *WIAT-II Numerical Operations Test* (Wechsler, 2005) was given. The first items measure number knowledge (i.e., identifying numbers, number sequence and transcoding) followed by written calculation problems involving addition, subtraction, multiplication and division of increasing difficulty (starting with single digit calculations followed by multi-digit calculations) (α = 0.88). To assess arithmetic fluency, children completed as many single digit additions/subtractions as possible within one minute (max = 30 per subtest). All operands and answers were below 20. Items 1 to 20 include only single digits as operands and answers (e.g., addition: 2 + 5; subtraction: 7 − 3), items 21 to 30 involve crossing the decade (e.g., addition: 5 + 7; subtraction: 14 − 6). The number of correctly solved items per second (efficiency) was calculated for each subtest. Test-retest reliability: Addition 0.92 and Subtraction 0.88.

## Results

Table 1 shows descriptive statistics across measures for the three groups (Typical reader, Dyslexia, Dyslexia + DLD) together with effect sizes (Cohen’s *d*) for the group comparisons.

**Table 1:** Performance of children from the typical, dyslexia and dyslexia + DLD groups at follow-up together with effect sizes for group differences

	Typical		Dyslexia		Dys + DLD		TD vs. Dyslexia	TD vs. DLD	Dys vs. Dyslexia + DLD
	Mean	SD	Mean	SD	Mean	SD	Effect size (d) [CIs]		
ExpVocab	52.31	5.86	50.28	7.93	37.50	7.72	0.33 [−1.6; 0.83]	2.39 [1.91; 2.86]	1.64 [0.95;2.31]
RecVocab	111.67	13.26	106.44	12.38	88.71	10.21	0.40 [−0.01;0.89]	1.79 [1.34; 2.23]	1.60 [0.91; 2.27]
ListenComp	14.71	2.77	13.39	2.93	8.25	4.11	0.47 [−0.02; 0.96]	2.14 [1.67; 2.60]	1.39 [0.72;2.04]
PhonDel	19.67	3.19	13.44	4.85	11.19	5.26	1.83 [1.3; 2.35]	2.37 [1.88;2.85]	0.44 [−0.16; 1.04]
RANdigits	2.16	0.47	1.48	0.35	1.55	0.45	1.48 [0.96; 2.0]	1.32 [0.88; 1.75]	−0.15 [−0.75;0.45]
NWrep	11.78	2.27	10.78	2.29	8.54	3.86	0.44 [−0.05;0.93]	1.25 [0.82; 1.68]	0.67 [0.06;1.28]
TOWREwds	114.77	9.94	92.28	14.72	85.50	16.83	2.13 [1.59;2.67]	2.59 [2.09; 3.07]	0.42 [−0.18; 1.02]
TOWRE nonwds	115.13	12.60	90.22	11.63	87.93	14.04	1.99 [1.45;2.53]	2.51 [2.02; 2.99]	0.17 [−0.42; 0.77]
Reading (Std Score)	111.30	10.80	87.56	10.71	82.68	9.63	2.20 [1.65; 2.74]	2.69 [2.20; 3.19]	0.48 [−0.12; 1.08]
Regular Words	27.20	2.24	20	5.11	16.21	7.20	2.67[2.10;3.24]	3.11 [2.59; 3.63]	0.58 [−0.02; 1.19]
Exception Words	25.51	2.78	17.78	6.56	13.32	6.39	2.29 [1.73; 2.83]	3.38 [2.84; 3.92]	0.69 [0.08; 1.30]
Nonwords	23.31	4.76	11.78	5.64	9.96	6.81	2.37 [1.81; 2.92]	2.60 [2.10; 3.08]	0.28 [−0.31; 0.88]
ReadComp	112.56	10.12	109.35	9.14	95.87	10.66	0.32 [−0.18; 0.82]	1.64 [1.16; 2.11]	1.34 [0.64; 2.03]
ArithSkills	109.33	15.70	89.83	12.95	80.79	15.17	1.26 [0.75; 1.76]	1.83 [1.38; 2.27]	0.63 [0.02; 1.23]
PIQ	109.12	12.36	102.00	11.08	90.69	13.76	0.58 [0.12;1.04]	1.46 [1.03;1.89]	0.89 [0.30;1.47]

Comparing the Dyslexia with the Typical Reader group, in line with the identification criteria, the Dyslexia group fall some 2SD below the typical readers on both timed and untimed reading measures but the effect size for the group difference in reading comprehension is small (d = 0.32) and not significant (see the left most column of effect sizes). The Dyslexia group performed more poorly on expressive and receptive vocabulary measures and listening comprehension - again effect sizes were small and not significant. They showed large deficits in phoneme awareness and RAN digits, in line with their dyslexia profile; there was a medium effect size for nonword repetition (d = 0.44) which was not significant (arguably because of the relatively small sample size). The Group with Dyslexia + DLD (centre column of effect sizes) had a more even profile, with large group differences across language and reading measures relative to Typical Readers (ds > 2) including in reading comprehension. Performance on phonological measures (phoneme awareness RAN digits and nonword repetition) was also impaired (ds > 1). Both dyslexia groups were impaired in the tasks assessing mathematical skills and similarly, there was a stepwise pattern in Performance IQ: Typical Reader > Dyslexia > Dyslexia + DLD. Differences between the two dyslexia groups are summarized in the far right column of Table 1. In line with identification criteria, the groups differed on language measures and the Dyslexia + DLD groups were generally weaker except in tasks tapping phonological skills: subgroup differences in phoneme deletion, RAN and in nonword reading were small and not significant.

Next to evaluate the research questions, we formed composite scores reflecting performance in (i) Language (ii) Phonology (iii) Reading (Word level) and (iv) Arithmetic. Composite scores are more reliable than individual measures. The Language Composite comprised performance in expressive vocabulary, receptive vocabulary and listening comprehension. The Phonology Composite comprised performance in phoneme deletion, RAN digits and nonword repetition, The Reading Composite comprised performance in words and nonword fluency (TOWRE), and untimed reading of regular, irregular and nonwords. Finally the Arithmetic Composite comprised untimed performance in numerical operations and one-minute tests of addition and of subtraction fluency. In each case, we standardized the scores of the contributing measures across the whole sample and then averaged the z-scores.

To assess group differences across the four domains we next conducted a series of ANOVAs followed by tests of group differences in marginal means using the Holm correction. There were group differences on all composite measures: Language F(2,187) = 66.22; Phonology F(2,185) = 47.30; Reading F(2,187) = 100.05; Arithmetic F(2,186) = 61.31; *p* <.001 for each composite. The groups also differed in reading comprehension: F(2,181) = 41.99. The differences between the Dyslexia + DLD group and the typical readers was highly significant across all composites (*p* <.0000). The Dyslexia + DLD group also performed less well than the Dyslexia group in Language, Reading, Arithmetic (*p* =.04) and Reading Comprehension but the Group difference was not significant on the Phonology composite (*p* =.06). Finally, as expected, the Dyslexia group performed significantly less well than the Typical group in Reading and Phonology and also in Language (*p* =.038); the group difference was not significant for Reading Comprehension (*p* =.29).

**Fig. 1:**
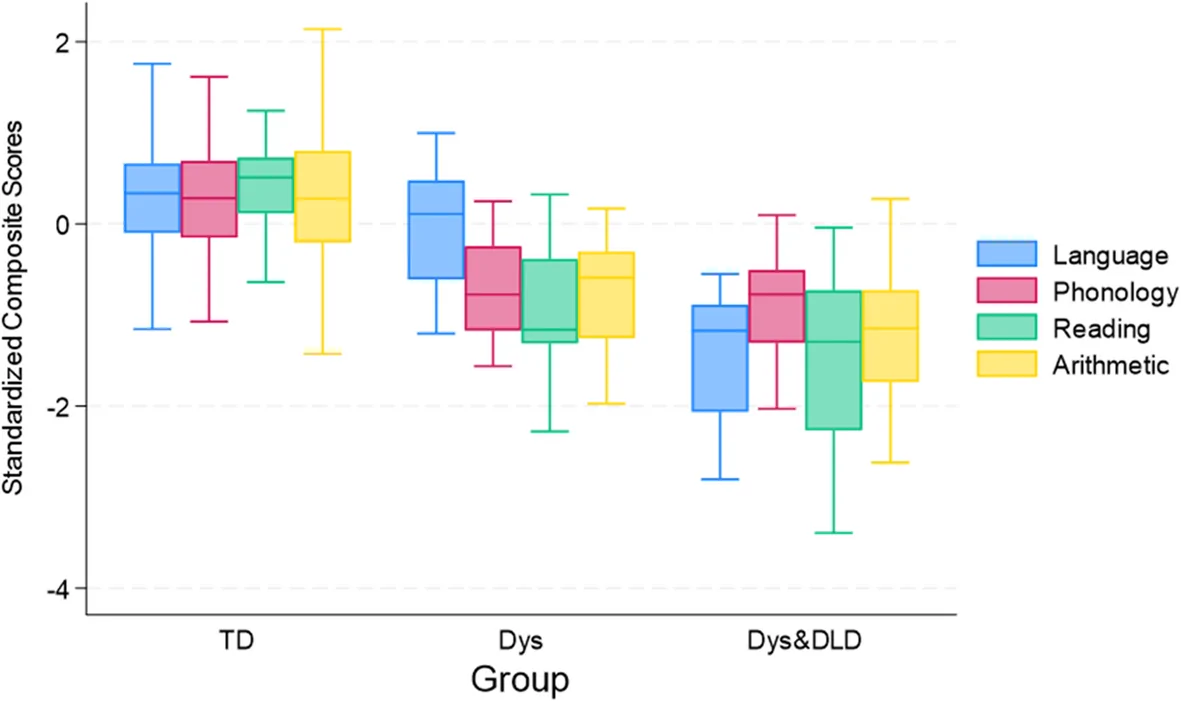
Box plot showing the performance of the Typical reader, Dyslexia and Dyslexia+DLD groups in Language, Phonology, Reading and Arithmetic (Compsite Scroes)

The findings of these analyses on composite scores are shown in Fig. 1, with effect sizes for the comparisons in Table 2. These data highlight the fact that, although the Group with Dyslexia does not reach criteria for DLD they do have significant language impairments (g = 0.52) and their phonological skills are weak. Arithmetic attainment is also significantly poor. By comparison the Dyslexia + DLD have very severe deficits (> 2SD below the Typical group), performing less well than the Dyslexia-only group in reading and marginally less well in arithmetic.

**Table 2:** Effect sizes of group differences on Language, Phonology, Reading and Arithmetic Composite Measures (Hedges *g*)

	**TD vs Dyslexia**	**TD vs Dyslexia + DLD**	**Dys vs Dyslexia + DLD**
Composite Scores	Effect size (g) [CIs]		
Language	0.52 [.02; 1.01]	2.82 [2.31; 3.32]	1.96 [1.24;2.67]
Phonology	1.78 [1.25;.2.30]	2.14 [1.67; 2.62]	.42 [-.17; 1.02]
Reading	2.70 [2.13; 3.27]	3.23 [2.70; 3.76]	.54 [.-.07;1.19]
Arithmetic	1.41 [.90;1.92]	2.00 [1.53;2.46]	0.65 [.04; 1.24]

Finally, there were strong correlations between language and phonology composites (*r* =.495); performance in Reading and Arithmetic was also highly correlated (*r* =.72).

## Discussion

We used data from a longitudinal study of children at risk of poor reading to examine the similarities and differences in language and literacy profiles between children who at age 8 were classified as dyslexic, comparing those with or without co-occurring developmental language disorder (DLD). First, one year after the children were classified, we showed, that, although the Dyslexia-only group did not reach criteria for DLD, they did experience subclinical language impairments. In fact, of the group */18 had below average standard scores on tests of receptive or expressive vocabulary.

Second, as expected, both dyslexia groups experienced significant difficulty on phonological tasks. Interestingly, their profiles were somewhat different with the Dyslexia + DLD group having less difficulty on RAN and more difficulty on the nonword repetition task. This profile is reminiscent of the differences posited by Ramus et al. (2013) Isee also (see also Bishop et al., 2009) whereby children with DLD have impaired phonological representations whereas children with Dyslexia primarily have problems in operating on them (Mundy & Carroll, 2012; for similar arguments). Third, in line with views regarding the stability of reading skills, the two dyslexia groups continued to experience problems with word-level decoding in reading; in contrast, it was only the Dyslexia + DLD group that experienced significant reading comprehension difficulties, as previously reported (Snowling et al., 2020).

We now turn to consider the theoretical, clinical and educational implications of these findings.

Together, they accord with views that dyslexia as a language learning impairment. Moreover, there is increasing evidence that, rather than considering phonological and non-phonological skills as independent, as implied by the 2D-model of the relationship between dyslexia and DLD, they are strongly correlated, with 25% shared variance in the present study. Earlier in development we propose there is an even stronger relationship, such that language can be viewed as a unitary factor which becomes more differentiated as learning to read begins to have a reciprocal influence on phonological skills (Snowling & Hulme 2025). Notwithstanding this, not all children with DLD have reading impairments. Although we did not examine the performance of such children in the present study, we have previously reported that early in development they have weak phonology but these difficulties decrease over time being less evident in phoneme awareness and RAN tasks. Others have shown that as a group they do not show RAN deficits consistent with the view that their impairments are a component of language development rather than on tasks that tap retrieval process. In this study, Dyslexia + DLD group is thus the outcome of two risks factors that are additive: one affecting the development of oral language (a marker of which is poor nonword repetition; Conti-Ramsden et al., 2001); the other affecting phonological output processing - a possible endophenotype of dyslexia (Snowling & Melby-Lervag, 2016).

Turning to clinical implications, we agree with Stevens et al. (2022) that the language difficulties in dyslexia have been underplayed. In the present study, both groups fulfilled criteria for dyslexia. Had oral language skills not been assessed then the comorbid group would not have their educational needs properly served. It follows that assessments of children with reading difficulties should include some measure of oral language skill and particularly so if the child is failing to respond to Tier 2 reading instruction (Duff et al., 2008). More generally, universal screening within the MTSS system should include language along with reading measures if intervention is to be appropriately planned.

It is also interesting to consider the role that a reciprocal relationship between spoken language and reading might play over time. In the present study, as in previous work, we have mainly focused on early language as a key precursor of reading – in this case, the language deficits associated with DLD as an additional risk factor for reading difficulties. However, in older children, reading becomes an important source of input for broader language development because of the particular properties of written text (Nation et al., 2022): through reading, children are exposed to vocabulary, syntactic and narrative structures and abstract and decontextualized language that is richer than much of what they encounter in spoken conversation. Although it is not something we have tested directly in the current study, it is possible that the moderate language deficit in our Dyslexia-only group partially reflects the reduced language input they are receiving from text relative to their peers, rather than being solely a consequence of (earlier) intrinsic weaknesses in spoken language. The practical implication of this would underscore the importance of enriching children’s language input (for example, through audiobooks) alongside supporting basic language and literacy skills.

## Conclusions

Note: dyslexics experience comorbid motor and EF deficits (d = 0.4) but these are greater in the DLD + dyslexia groups.

## Supplementary Information

Below is the link to the electronic supplementary material.


Supplementary Material 1 (DOCX 84.0 KB)


## References

[CR1] Adlof, S. M., & Hogan, T. P. (2018). Understanding dyslexia in the context of developmental language disorders. *Language, Speech, and Hearing Services in Schools,**49*(4), 762–773. 30458538 10.1044/2018_LSHSS-DYSLC-18-0049 PMC6430503

[CR2] Bishop, D. V., & Adams, C. (1990). A prospective study of the relationship between specific language impairment, phonological disorders and reading retardation. *Journal of Child Psychology and Psychiatry,**31*(7), 1027–1050. 2289942 10.1111/j.1469-7610.1990.tb00844.x

[CR3] Bishop, D. V., McDonald, D., Bird, S., & Hayiou-Thomas, M. E. (2009). Children who read words accurately despite language impairment: Who are they and how do they do it?. Child development, 80(2), 593-605. 10.1111/j.1467-8624.2009.01281.x} PMC2805876 19467013

[CR4] Bishop, D. V., & Snowling, M. J. (2004). Developmental dyslexia and specific language impairment: Same or different? *Psychological Bulletin,**130*(6), 858. 15535741 10.1037/0033-2909.130.6.858

[CR5] Brownell, R. (2000). *Expressive one-word picture vocabulary test: Manual*. Academic Therapy.

[CR6] Catts, H. W., Adlof, S. M., Hogan, T. P., & Weismer, S. E. (2005). Are specific language impairment and dyslexia distinct disorders? *Journal of Speech, Language, and Hearing Research*. 10.1044/1092-4388(2005/096) PMC2853030 16478378

[CR7] Duff, F. J., Fieldsend, E., Bowyer‐Crane, C., Hulme, C., Smith, G., Gibbs, S., & Snowling, M. J. (2008). Reading with vocabulary intervention: Evaluation of an instruction for children with poor response to reading intervention. *Journal of Research in Reading, 31*(3), 319–336. 10.1111/j.1467-9817.2008.00376.x

[CR8] Conti-Ramsden, G., Botting, N., Simkin, Z., & Knox, G. (2001). Follow-up of children attending infant language units: Outcomes at 11 years of age. *International Journal of Language & Communication Disorders,**36*(2), 207–219. 11344595

[CR9] Duff, F. J., Fieldsend, E., Bowyer‐Crane, C., Hulme, C., Smith, G., Gibbs, S., & Snowling, M. J. (2008). Reading with vocabulary intervention: Evaluation of an instruction for children with poor response to reading intervention. Journal of Research in Reading, 31(3), 319-336. 10.1111/j.1467-9817.2008.00376.x}

[CR10] Forum for Research in Literacy and Language. (2012). *Diagnostic test of word reading processes*. GL Assessment.

[CR11] Hulme, C., Nash, H. M., Gooch, D., Lervåg, A., & Snowling, M. J. (2015). The foundations of literacy development in children at familial risk of dyslexia. Psychological science, 26(12), 1877-1886. 10.1177/0956797615603} PMC4676358 26525072

[CR12] Göbel, S. M. (2014). ‘Number Processing and Arithmetic in Children and Adults with Reading Difficulties’, in Roi Cohen Kadosh, and Ann Dowker (Eds.), *The Oxford Handbook of Numerical Cognition*, Oxford Library of Psychology (2015; online edn, Oxford Academic, 3 Mar. 2014). 10.1093/oxfordhb/9780199642342.013.044

[CR13] Joanisse, M. F., Manis, F. R., Keating, P., & Seidenberg, M. S. (2000). Language deficits in dyslexic children: Speech perception, phonology, and morphology. *Journal of Experimental Child Psychology*, *77*(1), 30–60. 10964458 10.1006/jecp.1999.2553

[CR14] McArthur, G. M., Hogben, J. H., Edwards, V. T., Heath, S. M., & Mengler, E. D. (2000). On the “specifics” of specific reading disability and specific language impairment. *Journal of Child Psychology and Psychiatry,**41*(7), 869–874. 10.1111/1469-7610.00674 11079429

[CR15] Mundy, I. R., & Carroll, J. M. (2012). Speech prosody and developmental dyslexia: Reduced phonological awareness in the context of intact phonological representations. *Journal of Cognitive Psychology*, *24*(5), 560–581.

[CR16] Nash, H. M., Hulme, C., Gooch, D., & Snowling, M. J. (2013). Preschool language profiles of children at family risk of dyslexia: Continuities with specific language impairment. Journal of Child Psychology and Psychiatry, 54(9), 958-968. } 10.1111/jcpp.12091} PMC4523580 23772651

[CR17] Nation, K., Dawson, N. J., & Hsiao, Y. (2022). Book language and its implications for children’s language, literacy, and development. *Current Directions in Psychological Science*. 10.1177/09637214221103264

[CR18] Perfetti, C., & Stafura, J. (2014). Word knowledge in a theory of reading comprehension. *Scientific Studies of Reading*, *18*(1), 22–37.

[CR19] Ramus, F., Marshall, C. R., Rosen, S., & Van Der Lely, H. K. (2013). Phonological deficits in specific language impairment and developmental dyslexia: Towards a multidimensional model. *Brain,**136*(2), 630–645. 23413264 10.1093/brain/aws356 PMC3572935

[CR20] Rutter, M., & Yule, W. (1975). The concept of specific reading retardation. *Journal of Child Psychology and Psychiatry*, *16*(3), 181–197. 1158987 10.1111/j.1469-7610.1975.tb01269.x

[CR21] Scarborough, H. S. (1998). Predicting the future achievement of second graders with reading disabilities: Contributions of phonemic awareness, verbal memory, rapid naming, and IQ. *Annals of Dyslexia*, *48*, 115–136.

[CR22] Snowling, M., Bishop, D. V. M., & Stothard, S. E. (2000). Is preschool language impairment a risk factor for dyslexia in adolescence?. *The Journal of Child Psychology and Psychiatry and Allied Disciplines, 41*(5), 587–600. 10.1111/1469-7610.00651 10946751

[CR23] Snowling, M. J., Duff, F. J., Nash, H. M., & Hulme, C. (2016). Language profiles and literacy outcomes of children with resolving, emerging, or persisting language impairments. Journal of Child Psychology and Psychiatry, 57(12), 1360-1369. 10.1111/jcpp.12497} PMC5132029 26681150

[CR24] Snowling, M. J., & Melby-Lervåg, M. (2016). Oral language deficits in familial dyslexia: A meta-analysis and review. *Psychological Bulletin,**142*(5), 498. 26727308 10.1037/bul0000037 PMC4824243

[CR25] Snowling, M. J., Hayiou-Thomas, M. E., Nash, H. M., & Hulme, C. (2016). Dyslexia and Developmental Language Disorder: comorbid disorders with distinct effects on reading comprehension. Journal of Child Psychology and Psychiatry, 61, 672-680. 10.1111/jcpp.13140} PMC7317952 31631348

[CR26] Snowling, M. J., Nash, H. M., Gooch, D. C., Hayiou-Thomas, M. E., Hulme, C., & Wellcome Language and Reading Project Team. (2019). Developmental outcomes for children at high risk of dyslexia and children with developmental language disorder. *Child development, 90*(5), e548– e564. 10.1111/cdev.13216 PMC6767399 30676649

[CR27] Snowling, M. J., Hayiou‐Thomas, M. E., Nash, H. M., & Hulme, C. (2020). Dyslexia and developmental language disorder: Comorbid disorders with distinct effects on reading comprehension. *Journal of Child Psychology and Psychiatry, 61*(6), 672–680. 10.1111/jcpp.13140 PMC7317952 31631348

[CR28] Snowling, M.J., & Hulme, C. (2025). The Reading Is Language Model: A Theoretical Framework for Language and Reading Development and Intervention. *Annual Review Developmental Psychology. 7*:195–218. 10.1146/annurev-devpsych-111323-084821

[CR29] Stanovich, K. E., & Siegel, L. S. (1998). The role of IQ in the diagnosis of reading disorders: The quest for a subtype based on aptitude/achievement discrepancy. In J. Rispens, T. A. Yperen, & W. Yule (Eds.), *Perspectives on the classification of specific developmental disorders* (pp. 105–136). Springer Netherlands.

[CR30] Stevens, E. A., Hall, C., & Vaughn, S. (2022). Language and reading comprehension for students with dyslexia: An introduction to the special issue. *Annals of Dyslexia*, *72*(2), 197–203. 35587302 10.1007/s11881-022-00260-6

[CR31] Torgesen, J. K., Rashotte, C. A., & Wagner, R. K. (1999). *TOWRE: Test of word reading efficiency*. Pro-ed.

[CR32] Vellutino, F. R. (1979). *Dyslexia: Theory and research*. The MIT Press.

[CR33] Wechsler, D. (2005). *Wechsler Individual Achievement Test 2nd UK Edition (WIAT II)*. The Psychological Corporation.

[CR34] Wiig, E. H., Semel, E., & Secord, W. A. (2006). *Clinical Evaluation of Language Fundamentals 4th Edition (CELF-4).* Pearson.

