# Supplementary material for: Dyslexia with and without developmental language disorder: Profile analysis
Source: Ann Dyslexia. 2026 Mar 3;76(2):267–78. doi: 10.1007/s11881-025-00358-7 (PMC13529876; doi:10.1007/s11881-025-00358-7)
Supplement: Supplementary file 1 [file 11881_2025_358_MOESM1_ESM.docx]

*Appendix*

*Participant Flow through Wellcome Language and Reading Study*
